# Progress in Surgery

**Published:** 1899-04-08

**Authors:** 


### Progress in Surgery.

### GENERAL SURGERY.

(*Continued from page 11.*)

**Fractures and Dislocations (Upper Extremity)** *continued.*—In opening the recent discussion on injuries of the elbow joint at Edinburgh, Dr. E. H. Bennett[28] presented the cast of a limb with compound separation, the diaphysis being thrust through the skin and protruded some two inches, stripped of its periosteum. The brachial artery was torn through and the wound suppurated freely, but the elbow joint remained intact and preserved its movements almost without limitation. Dr. C. H. Frazier[29] demonstrates the advantages of treating fractures of the elbow joint by acute flexion, and quotes 250 cases of severe injury to this joint treated by Robert Jones of Liverpool in 1892-94, as being unquestionably successful. A case of severe compound comminuted fracture of the lower end of the humerus with incomplete rupture of the median nerve is described by Mr. H. F. Waterhouse.[30] It occurred in a little girl who was thrown from a goat chaise nine months previously. A skiagram showed that the injury to the nerve was caused by the sharp anterior margin of the lower end of the upper fragment. Although the elbow joint presented an extraordinary appearance the movements were almost perfect; extension, pronation, and supination were perfect, and flexion only slightly limited; but there was some atrophy of the muscles of the forearm, coldness of part of the hand, and ulcers on the tips of the index and middle fingers. The good movement he attributed to early (seventh day) passive movement. In fractures near the joints he advises massage from the first day—first very gently once a day, and afterwards twice a day. He lays down a rule, "Do not mind so much about the accurate apposition of the bony fragments in any fracture very near a joint, but think rather of the subsequent mobility." Dr. G. H. Williams[31] relates an instance of greenstick fracture of both bones of the forearms in a child eighteen months old, which he had seen four weeks after the accident—a fall out of a low swing. After skiagrams had been taken, the bones of the left forearm were found to be most deformed, and therefore refractured under chloroform and put up in wooden splints. A fortnight later the right forearm was refractured and put up in the same manner. Both forearms were subsequentto the refracture placed in a silicate of soda splint, and the bones afterwards united in good position. A case of compound fracture of the forearm in a girl 10 years of age is recorded by Mr. W. S. Wright.[32] Acute traumatic gangrene of the hand and forearm ensued within 24 hours, and spread so rapidly that on the second day amputation of the arm was performed, and antistreptococcic serum injected. The favourable progress of the stump and the ultimate recovery of the patient were attributed in great measure to the injections. Dr. E. R. Colson[33] describes a rare case of excessive bone atrophy complicating an ununited fracture in both forearms of the same individual. A widow, aged 70, had ten years previously fallen on her left forearm, fracturing both bones, and was treated at a hospital with a splint for a month, when non-union was discovered. Six months before coming under observation paralysis agitans developed, but was confined to the left side. The X rays revealed the fact that both bones on the left side were atrophied to points, while the right ulna was in the same condition. Dr. Colson thought that there was no relation between the bone atrophy and the paralysis agitans. Dr. G. H. Edington[34] furnishes a sketch of an interesting specimen of comminuted fracture of the lower end of the radius. There was a transverse comminuted fracture about 1·25 cm. above the articular surface anteriorly and 3 cm. posteriorly, and also externally above the styloid process. The lower fragment was attached to the ulna by the inferior radio-ulnar ligaments, and also to the posterior surface of the radial shaft by the periosteum flooring the grooves for the extensor tendons. Anteriorly the line of fracture while mainly transverse presented a sinuosity. The specimen was taken post-mortem, together with that of the fractured humerus above mentioned, from a man who had fallen a distance of about twenty feet.

[28] Brit. Med. Jour., Oct. 29, 1898, p. 1317. [29] Amer. Med. Mag., Vol. X., No. 7, p. 4002; Amer. Med. Surg. Bull., Aug. 10, 1898, p. 726. [30] Clin. Jour., Sept. 7, 1898, p. 381. [31] New York Med. Rec., Sept. 24, 1898, p. 455. [32] Lancet, Aug. 13, 1898, p. 407. [33] Annals of Surgery, Oct., 1898, p. 503. [34] Glasgow Med. Jour., Aug., 1898, p. 119.

